# Response of tear cytokines following intense pulsed light combined with meibomian gland expression for treating meibomian gland dysfunction-related dry eye

**DOI:** 10.3389/fendo.2022.973962

**Published:** 2022-09-15

**Authors:** Haozhe Yu, Weizhen Zeng, Gezheng Zhao, Jing Hong, Yun Feng

**Affiliations:** Department of Ophthalmology, Peking University Third Hospital, Beijing, China

**Keywords:** meibomian gland dysfunction, intense pulsed light, cytokine, tear, MGD, IPL

## Abstract

**Purpose:**

This study compared the changes in tear inflammatory cytokine levels after intense pulsed light (IPL) combined with meibomian gland expression (MGX) (IPL group) and instant warm compresses combined with MGX (physiotherapy group) as treatments for meibomian gland dysfunction (MGD)-related dry eye disease (DED) to explore their similarities and differences in therapeutic mechanisms.

**Methods:**

This study was a *post-hoc* analysis of a randomized controlled trial. Thirteen patients with MGD-related DED were enrolled in each group and received three treatments correspondingly with 3-week intervals. The levels of 20 tear cytokines, namely, TNF-α, IL-6, MMP-9, CXCL8/IL-8, CXCL10/IP-10, IL-10, EGF, IL-6R, IL-1β, IFN-γ, lactoferrin, Fas ligand, IL-17A, LT-α, S100A9, LCN2/NGAL, IL-13, IL-12/IL-23p40, Fas, and CCL11/Eotaxin, were measured at baseline, before the second and third treatments, and 3 weeks after the third treatment. The primary outcome was the difference in cytokine levels between baseline and the last measurement, and the trends were analyzed at each measurement point.

**Results:**

At the last measurement, a significant decrease was observed in all tear cytokines for both IPL and physiotherapy groups compared with baseline. The IPL group showed greater reductions in IL-6, IL-6R, IL-1β, IL-13, and CCL11/Eotaxin than the physiotherapy group. TNF-α, CXCL8/IL-8, CXCL10/IP-10, IL-10, EGF, IL-1β, IFN-γ, and Lipocalin-2/NGAL levels continued to decrease with treatment time. Important interactions were found in the changes of IL-6 and IL-13 levels, where the levels first decreased and then slightly increased in the physiotherapy group after treatment, while they continued to decrease in the IPL group.

**Conclusions:**

The mechanisms of IPL and physiotherapy in treating MGD-related DED were both associated with reducing inflammation, and the superiority of IPL could be attributed to its better inhibitory effect on inflammatory cytokines like IL-6. In addition, several cytokines were on a downward trend during treatment, suggesting that the vicious cycle of DED was suppressed.

## Introduction

Dry eye disease (DED) has been recognized as a high incidence of ocular surface disease over the past few decades with its estimated prevalence of nearly 12% around the world, and the trend continuously increases rapidly (1, 2). In clinical practice, DED can be divided into two types: aqueous-deficient due to low tear production and evaporative due to meibomian gland dysfunction (MGD). The latter is the most common type, with more than 80% of patients belonging to this type (3). MGD usually presents as an abnormal quantity and quality of secreted meibum, mainly caused by epithelial hyperkeratinization and high viscosity of meibomian lipids, which will further lead to ongoing chronic inflammation, resulting in meibomian gland dropout and atrophy (4, 5). In this case, the stability of the lipid layer of the tear film will decrease, thereby exhibiting high evaporation and hyperosmolarity of the tear (6, 7).

Many therapies targeted to the lipid composition and chronic inflammatory state of meibomian glands have been proposed including artificial tears, omega-3 supplements, anti-inflammatories like cyclosporine A, and antibiotics like doxycycline (8, 9). Recently, non-pharmaceutical interventions for MGD including warm compress, manual physical expression, and intraductal probing which act directly on meibomian glands have attracted more and more attention with their favorable clinical effect (10). Of these, intense pulsed light (IPL) treatment based on non-laser and high-intensity light sources is considered to be a possible better option (11, 12). In addition, several randomized clinical trials reported a better improvement of combination therapy of IPL with other approaches such as meibomian gland expression (MGX), which therefore has been widely used in clinical practice nowadays (13–15).

Several possible mechanisms have been proposed to explain the symptomatic improvement of MGD and DED after IPL treatment, including inhibition of bacteria growth in the eyelid, reduction of viscosity in the meibum through heating, and dilation and reconstruction of meibomian gland ducts through electromagnetic waves (16). However, their exact mechanisms are still unclear and controversial due to a lack of evidence at the molecular level; clinical studies reported different correlations between decreased levels of inflammatory cytokines and improvement in MGD (17, 18). The photothermal effect of IPL on meibomian glands has not been confirmed by *in-vivo* experiments. Li et al. (19) further proposed neurogenic mechanisms on this basis. In addition, a recent study found that although IPL could activate fibroblasts and immune cells, it might also skew the metabolic pathway of targeted cells toward a glycolytic phenotype, thereby promoting oxidative stress and apoptosis (20). Such results affect the assessment of the safety and efficacy of IPL and limit its further application (21, 22). On the contrary, instant warm compresses are considered to be a conservative measure that was worth promoting with excellent safety although its mechanism is also not well understood (23, 24). Nowadays, the multiplex assay of cytokines in tears has been recognized as an effective means to explore the mechanism of ocular surface diseases such as DED and keratoconus as well as monitor their progress of treatment (25, 26). In this study, we explored the interaction of the IPL and common physical therapy with the ocular surface by comparing their changes in tear cytokine profiles based on our previous randomized control trial, with a view to providing a reference for the mechanistic interpretation and choice of therapy for MGD.

## Method

### Study design and subjects

This is a *post-hoc* analysis based on our previous randomized controlled trial which aimed to compare the efficacy of IPL and instant warm compresses in combination with MGX in improving DED. The study was registered on the Chinese Clinical Trial Registry (ChiCTR1800014787), and its detailed design and primary outcome have been reported elsewhere (15). In short, 120 patients with DED caused by MGD from four centers were randomized to receive either IPL + MGX or instant warm compresses + MGX therapy, and each subject was treated three times at 3-week intervals. All subjects had no additional drug interventions other than the use of artificial tears three times a day. The clinical parameters considered mainly included tear film break-up time (TBUT), self-evaluated Standard Patient Evaluation of Eye Dryness (SPEED) questionnaire score, meibomian gland yielding secretion score (MGYSS), and corneal staining score, and the primary outcome was the improvement of tear break-up time of the right eye at 3 weeks after the last treatment compared to baseline.

In the current study, 26 patients from a single center at Peking University Third Hospital were included with a 1:1 ratio of IPL + MGX (IPL) to instant warm compresses + MGX (physiotherapy) groups. Their tears were collected at different time points (baseline and before/after treatment), which was consistent with previous randomized trials, and the cytokine levels of their tears were measured. The primary endpoint was the change in cytokine levels of tears at 3 weeks after the third therapy from baseline. The study was approved by Peking University Third Hospital Medical Science Research Ethics Committee and conducted under the guidance of the Declaration of Helsinki. All subjects signed an informed consent.

### Tear collection and cytokine concentration measurement

The subjects’ tears were collected at 0 to 14 days before the first treatment (baseline), before the second (first repeated measurement) and third interventions (second repeated measurement), and 3 weeks after the third treatment (third repeated measurement). For each time point, a total of 6.6 μl of basal tear samples were collected carefully from the lower lateral tear meniscus of each eye of the subjects using 2.2 μl of microcapillaries. The tears from the different patients and eyes were independently transferred to 0.5-ml microcentrifuge tubes and stored at −80°C until further analysis.

The concentration of a set of 20 cytokines, namely, tumor necrosis factor α (TNF-α), interleukin 6 (IL-6), matrix metalloproteinase-9 (MMP-9), interleukin 8 (CXCL8/IL-8), chemokine C-X-C ligand 10 (CXCL10/IP-10), interleukin 10 (IL-10), epidermal growth factor (EGF), interleukin 6 receptor (IL-6R), interleukin 1 beta (IL-1β), interferon gamma (IFN-γ), lactoferrin, Fas ligand, interleukin 17A (IL-17A), lymphotoxin-alpha (LT-α), S100 calcium binding protein A9 (S100A9), Lipocalin-2 (LCN2/NGAL), interleukin 13 (IL-13), interleukin 12 (IL-12/IL-23p40), Fas, and chemokine C-C motif ligand 11 (CCL11/Eotaxin), was measured through a microsphere-based immunoassay according to the instructions of the manufacturers (17, 27).

### Statistical analysis

The statistical analysis was conducted using IBM SPSS 24.0 and R 4.0.4 software. The changes in the cytokine levels were presented as the ratio of cytokine levels at different time points compared to baseline for further repeated measurement analysis. The reductions in cytokine levels at baseline and last measurement in patients assigned to the IPL and physiotherapy groups, as well as their trends during ongoing treatment, were analyzed through generalized estimating equations based on the compound symmetric correlation matrix, respectively (28). The age, sex, SPEED score of patients as well as TBUT, fluorescein staining of the cornea, MGYSS score, and baseline tear cytokine levels were included in the model as covariates. The nominal *P <*0.05 was considered statistically significant.

## Results

**Figure 1:**
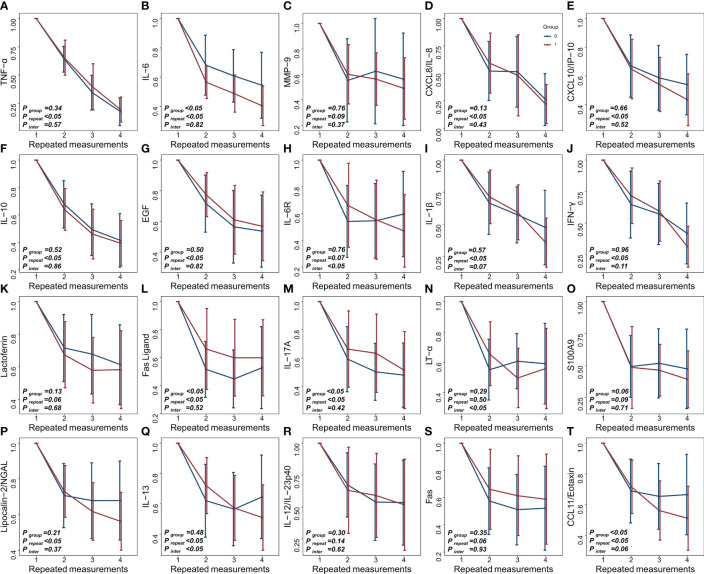
**(A–T)** Longitudinal changes in tear cytokine levels under different treatments (red: IPL group, blue: physiotherapy group).

**Figure 1** shows the changes in tear cytokine levels of patients during the three follow-up visits after IPL and physiotherapy. There were significant decreases in all tear cytokine levels at the last measurement after treatment. The IPL group had significantly greater decreases in IL-6, IL-6R, IL-1β, IL-13, and CCL11/Eotaxin than the physiotherapy group. A superior cytokine-lowering effect of physiotherapy was found for EGF, FasL, IL-17A, and Fas; however, these differences disappeared after adjusting for baseline covariates.

Repeated measures analysis further indicated that there were group differences in the effect of IPL and physiotherapy on decreasing IL-6 and CCL11/Eotaxin concentrations in tears, and their levels continued to decrease with treatment time. However, the interaction of CCL11/Eotaxin was marginally significant, suggesting that IPL was more effective than physiotherapy in reducing CCL11/Eotaxin, and their differences further increased over time. In addition, between-group and time-point differences were found for FasL and IL-17A, while there was no difference in the primary outcome, implying that these cytokine levels varied differently with time and reached similar levels at the last measurement.

Although there were no between-group differences, significant time effects were found in the decreases of TNF-α, CXCL8/IL-8, CXCL10/IP-10, IL-10, EGF, IL-1β, IFN-γ, and Lipocalin-2/NGAL, indicating that their levels continued to decline with treatment. A marginally significant interaction was also found for IL-1β, demonstrating a higher cytokine-lowering effect in the IPL group. No significant between-group and time effects were found for MMP-9, lactoferrin, IL-17A, S100A9, IL-12/IL-23p40, or Fas, suggesting that the lowering effects of these cytokines were similar between groups and did not change with prolonged treatment.

Furthermore, significant interaction effects were found for IL-6R, LT-α, and IL-13. IL-6R and IL-13 gradually decreased in the IPL group, while they first decreased and then increased in the physiotherapy group. LT-α levels in both groups tended to first decrease and then increase, but the increase in the IPL group was delayed compared with the physiotherapy group.

## Discussion

Several emerging therapies, such as IPL, have been proposed for MGD and its related DED; however, their superiority and specific mechanisms are still controversial. In the current study, we compared the changes in the levels of inflammatory cytokines in the tears of patients with MGD undergoing IPL treatment versus physiotherapy over time. Both the IPL and physiotherapy groups had reduced levels of all tested cytokines, and IPL demonstrated superior ability in reducing the levels of IL-6, IL-6R, IL-1β, IL-13, and CCL11/Eotaxin.

Proinflammatory cytokine levels in tears are closely related to the occurrence and development of MGD through a vicious inflammatory cycle, and many studies have tried to speculate on the mechanism of treatment of MGD through changes in these cytokines (29). Liu et al. (17) and Li et al. (30) reported significant decreases in the levels of IL-17A, IL-6, prostaglandin E2, CXCL1, CCL11, TNF-α, IFN-γ, IL-2, and TIMP-1 in the tears of MGD patients, suggesting that the therapeutic effect of IPL on MGD might be achieved by regulating inflammation. Our study further expanded the cytokine profile of the therapeutic mechanism of IPL. In this context, it is inappropriate to reveal the therapeutic mechanism of IPL or physiotherapy for MGD through only one or a few specific signaling pathways, while determining the interaction between signaling pathways and exploring their inflammatory cytokine networks through omics approaches might be promising (31, 32). In addition, we found that physiotherapy alone could also change the levels of inflammatory cytokines in tears, indicating that the combination of anti-inflammatory drugs or antibiotics with physiotherapy might not be necessary, thus avoiding the potential side effects of related drugs such as ocular hypertension (33).

IL-6 is an important signature molecule of MGD and MGD-related DED, which is associated with signs like meibography score, meibum quality, and Schirmer I test (34, 35). In addition, it also plays a key role in the pathological mechanisms by regulating lipid metabolism and the differentiation of Th17 cells that could induce severe DED (36, 37). IL-1β is thought to be involved in the vicious cycle of MGD because it could be recruited to the ocular surface by bone marrow-derived cells and further promote the secretion of more inflammatory cytokines by epithelial cells (38). Therefore, the better therapeutic effect of IPL on MGD and MGD-related DED might be related to its improved ability to reduce the levels of these cytokines. In addition, recent studies have reported the potential roles of IL-6R, IL-13, and CCL11/Eotaxin in autoimmune diseases such as primary Sjögren’s syndrome through T and B helper cells (39–43). Further exploration of the application of IPL for ocular surface symptoms of other immune-related diseases in this context is worth considering.

The levels of several cytokines that are generally thought to be anti-inflammatory were also reduced, such as IL-10 secreted by regulatory B cells (44–46). One possible reason for this is the duality of IL-10 in the ocular surface immune response to DED and MGD. For instance, Fukushima et al. (47) reported that IL-10 promoted the infiltration of eosinophils into the conjunctiva, and Roda et al. (48) found that IL-10 levels in tears were higher in DED patients. However, a previous study found no association between the reduction of IL-10 in tears after IPL with meibum expression improvement (18). In addition, IPL was shown to be unable to regulate IL-10 mRNA expression *in vivo*; therefore, its decrease might be affected by physiotherapy or a cascade reaction of inflammatory cytokines (49). Further exploration of the roles of such cytokines and their interactions in DED and MGD patients will help to reveal the mechanisms of therapeutic change.

There has been no agreement on the periods of treatment for MGD. It is generally accepted that long-term treatment for MGD is necessary when considering physiotherapy; however, there is considerable variation in the number and duration of IPL treatments, ranging from every few weeks or months to a single session (14, 50–53). Our current results show that during the treatment process, although some cytokines reached the lowest levels after the first treatment, there were still some cytokines whose concentration continued to decline, revealing the necessity for prolonged therapy to some extent. Most of these sustained response cytokines were involved in the vicious cycle of MGD and DED, such as TNF-α and IFN-γ, thereby reducing the inflammatory cascade reaction, which could also be used to explain the low levels of MMP-9, an inflammatory cytokine mainly secreted by corneal epithelial cells, after the first treatment.

It is worth noting that after treatment, the decrease in cytokine levels varied widely between patients, suggesting that the treatment benefit varied considerably among patients. Several studies have reported similar results, where the extent of meibomian gland dropout would greatly affect the patient’s response to treatment, and those patients with early-stage MGD were more suitable for IPL/MGX therapy; however, its mechanism remains unclear (54–56). In addition, MGX and instant warm compresses are not standardized clinical procedures, which can also affect a patient’s response to therapy. It has been proven that the type of meibum squeezed from MGX as well as the ambient temperature and type of equipment in warm compress therapy could affect the improvement of MGD (57, 58). Although almost all patients in this study demonstrated benefits from physiotherapy, Villani et al. (59) showed that physiotherapy was ineffective for nearly one-third of patients due to compliance and standardization. In this context, standardized IPL treatment procedures are recommended.

There are some limitations to this study. Although the current results were obtained through a *post-hoc* analysis of a rigorous prospective randomized controlled trial to avoid potential bias, the single-center sample size was relatively small. We also only reported nominal *P*-values that were not adjusted for multiple hypothesis testing because of the exploratory nature of this study; therefore, the results should be interpreted with caution. In addition, both experimental and control groups included MGX treatment, and while it is expected that its effects could be counteracted in clinical trials to reflect the true effects of IPL, it is difficult to rule out its potential interactions with IPL or instant warm compresses. Furthermore, the last time point in this study was set at 3 weeks after the third treatment, and a longer follow-up is still required to determine the long-term effect of IPL and physiotherapy on MGD and DED.

## Data availability statement

The raw data supporting the conclusions of this article will be made available by the authors, without undue reservation.

## Ethics statement

This study was reviewed and approved by Peking University Third Hospital medical science research ethics committee. The patients/participants provided their written informed consent to participate in this study.

## Author contributions

JH, YF, and HY designed the study. HY wrote the initial draft. WZ collated and organized the data from the clinical trial. WZ, GZ, and YF revised the manuscript. All the authors made a substantial contribution to the article and approved the final submitted version.

## Funding

This study was supported by grants from the National Natural Science Foundation of China (Nos. 81700799 and 82070926).

## Conflict of interest

The authors declare that the research was conducted in the absence of any commercial or financial relationships that could be construed as a potential conflict of interest.

## Publisher’s note

All claims expressed in this article are solely those of the authors and do not necessarily represent those of their affiliated organizations, or those of the publisher, the editors and the reviewers. Any product that may be evaluated in this article, or claim that may be made by its manufacturer, is not guaranteed or endorsed by the publisher.
